# miRNAs as Predictive Factors in Early Diagnosis of Gestational Diabetes Mellitus

**DOI:** 10.3389/fendo.2022.839344

**Published:** 2022-03-07

**Authors:** Ilona Juchnicka, Mariusz Kuźmicki, Magdalena Niemira, Agnieszka Bielska, Iwona Sidorkiewicz, Monika Zbucka-Krętowska, Adam Jacek Krętowski, Jacek Szamatowicz

**Affiliations:** ^1^ Department of Gynecology and Gynecological Oncology, Medical University of Bialystok, Bialystok, Poland; ^2^ Clinical Research Centre, Medical University of Bialystok, Bialystok, Poland; ^3^ Department of Gynecological Endocrinology and Adolescent Gynecology, Medical University of Bialystok, Bialystok, Poland

**Keywords:** gestational diabetes, miR-16-5p, miR-142-3p, miR-144-3p, epigenetics, serum profiling, biomarkers, miRNA

## Abstract

**Introduction:**

Circulating miRNAs are important mediators in epigenetic changes. These non-coding molecules regulate post-transcriptional gene expression by binding to mRNA. As a result, they influence the development of many diseases, such as gestational diabetes mellitus (GDM). Therefore, this study investigates the changes in the miRNA profile in GDM patients before hyperglycemia appears.

**Materials and Methods:**

The study group consisted of 24 patients with GDM, and the control group was 24 normoglycemic pregnant women who were matched for body mass index (BMI), age, and gestational age. GDM was diagnosed with an oral glucose tolerance test between the 24th and 26th weeks of pregnancy. The study had a prospective design, and serum for analysis was obtained in the first trimester of pregnancy. Circulating miRNAs were measured using the NanoString quantitative assay platform. Validation with real time-polymerase chain reaction (RT-PCR) was performed on the same group of patients. Mann-Whitney U-test and Spearman correlation were done to assess the significance of the results.

**Results:**

Among the 800 miRNAs, 221 miRNAs were not detected, and 439 were close to background noise. The remaining miRNAs were carefully investigated for their average counts, fold changes, p-values, and false discovery rate (FDR) scores. We selected four miRNAs for further validation: miR-16-5p, miR-142-3p, miR-144-3p, and miR-320e, which showed the most prominent changes between the studied groups. The validation showed up-regulation of miR-16-5p (p<0.0001), miR-142-3p (p=0.001), and miR-144-3p (p=0.003).

**Conclusion:**

We present changes in miRNA profile in the serum of GDM women, which may indicate significance in the pathophysiology of GDM. These findings emphasize the role of miRNAs as a predictive factor that could potentially be useful in early diagnosis.

## Introduction

Gestational Diabetes Mellitus (GDM) is one of the leading diseases during pregnancy. According to the newest edition of the International Diabetes Federation (IDF) Diabetes Atlas, GDM affected nearly 17 million live births in the last year (1). Extensive hormonal changes during pregnancy are one of the reasons for increased insulin resistance. For instant, the hyperestrogenemic state observed during pregnancy contributes to alterations in insulin sensitivity. Estrogen may bind directly to insulin or its receptors, making them unavailable for insulin (2). Furthermore, human placental lactogen (hPL) decreases maternal insulin sensitivity in order to provide the fetus with sufficient nutrition (3). When the insulin release is insufficient and a glucose-lowering response is not achieved, the risk of GDM development is high (4). Meta-analysis showed that the most relevant risk factors for GDM are high BMI and thyroid disease (5). Another risk factors are increased fasting glycemia in the first trimester of pregnancy, abdominal obesity, family history of diabetes mellitus, genetic factors, environmental factors including lifestyle and diet, comorbidities like polycystic ovary syndrome (PCOS) (6, 7). Combinations of several risk factors more confidently indicate women at high risk of developing GDM (8). Considering, that utility of risk factors, such as i.e first-trimester fasting blood glucose concertation is limited (9), it is essential to search for the most ideal non-invasive biomarker for early GDM detection or even a predisposition to develop GDM.

MiRNAs are a group of non-encoding RNA molecules of 19-22 nucleotides that play a key role in the regulation of post-transcriptional gene expression (10, 11). Notably, one miRNA has the ability to bind with many genes by recognizing the not-necessarily complementary sequence at the end of the 3’-untranslated region (3’UTR) of the target mRNA (12). In this way, endogenous miRNAs control the expression of many genes and influence the processes that take place in cells, such as cell metabolism, proliferation, DNA repair, and apoptosis. Furthermore, data suggest that extracellular miRNAs act as modulators during physiological and pathological processes by transferring information between cells (13). Depending on which gene that the miRNA impacts, it can be either a stimulator or a suppressor of a pathological state (14).

MiRNA is detectable in various biological fluids, such as blood, urine, tears, saliva, and cerebrospinal, amniotic, or synovial fluid (15). In contrast to other RNA molecules, an important feature of miRNA is their stability and resistance to external factors, such as RNAse (16). This is due to the form in which they occur in biofluids. MiRNA forms complexes with lipoproteins or proteins (17). Moreover, the protective effect may be a result of their encasement inside membrane structures like exosomes, microparticles, or apoptotic bodies (17, 18). It has also been shown that repeated cycles of freezing and thawing do not cause significant changes in miRNA content in the serum (19). These mechanisms and non-invasive collection mean that circulating miRNAs have good potential as a biomarker.

In recent years, there have been a number of reports on changes in miRNA expression in various diseases, including metabolic disorders. One of the ultimate purposes of most of the studies is finding miRNAs that could help with identifying pathological processes, estimate the success of a patient’s response to therapy (20), or support the identification of high-risk groups (21). Zhao et al. were some of the first to describe changes in the sera of pregnant women with GDM (22). Since that time, many scientists have focused on changes in miRNA expression in GDM, but the available data are not consistent. Thus, the purpose of this study was to compare the miRNA expression profile in a group of patients in the first trimester of pregnancy and GDM diagnosed in the second trimester of pregnancy with that of a healthy control group. Then, based on these results, we sought to identify potential biomarkers of early GDM diagnosis.

## Materials and Methods

### Study Population

Project included four meetings, in the first trimester (9-12 week), in the second (24-26 week), in the third trimester (34-37 week) and three months after delivery. During the first trimester of pregnancy, fasting venous blood samples were collected into S-Monovette Gel Clotting Activator tubes (Sarstedt, Numbrecht, Germany). After complete clotting and centrifugation, the serum to be used for miRNA analysis was separated, transferred into DNase- and RNase-free tubes (Eppendorf, Hamburg, Germany), and stored at -80°C until they were assayed. To diagnose GDM all patients underwent a 75g oral glucose tolerance test (OGTT) in the second trimester, between 24^th^ and 27^th^ weeks of pregnancy. GDM was diagnosed according to the World Health Organization (WHO) criteria (23). In the experiment the serum from the first trimester was examined while both groups revealed normoglycemia. The study group (GDM) (n=24) and control group with normal glucose tolerance (NGT) (n=24) were carefully matched for pre-pregnancy body mass index (BMI), age, and gestational age. Women with the history of GDM, stillbirth, childbirth with congenital anomalies, pregnancy-induced hypertension, preeclampsia, cholestasis, premature delivery, acute or chronic inflammation, multiple pregnancy, pre-existing glucose intolerance, and active smokers were excluded from the study. Written informed consent was obtained from each patient, and the study was approved by the local ethics committee (Medical University of Bialystok).

### Biochemical Methods

Plasma glucose concentrations were measured using an enzymatic method with hexokinase (Cobas C11, Roche Diagnostics Ltd, Switzerland), and the serum insulin level was evaluated by an immunoradiometric method (DiaSource Europe SA, Belgium) using a Wallac Wizard 1470 Automatic Gamma Counter (Perkin Elmer, Life Science, Turku, Finland). Glycated hemoglobin (HbA1c) was assayed by high-performance liquid chromatography (Bio-Rad D-10, Bio-Rad Laboratories, Hercules, USA). The homeostasis model assessment of insulin resistance (HOMA-IR) and homeostatic model assessment of β-cell function were calculated for all women in each trimester of pregnancy. Moreover, in the second trimester, insulin sensitivity was measured using the OGTT insulin sensitivity index of Matsuda and DeFronzo (ISI_OGTT_).

### miRNA Isolation

MiRNA was isolated using the miRNeasy Serum/Plasma Advanced Kit (Qiagen, Germany) by following the manufacturer’s protocol. The isolation method is based on the innovative spin-column separation method with a silica membrane. The use of this kit allows us to obtain miRNA of high quality and purity, which is necessary for the subsequent stages of the experiment. The content of miRNA in extracted samples was checked with a fluorometer (Qubit 3.0, Thermo Fisher Scientific, Waltham, USA).

### Nanostring Analysis

For miRNA profiling, we used NanoString technology with a digital color-coded barcode for direct and multiplex marking of target sequences of 800 miRNAs. The method uses about 50 nucleotide probes per 1 miRNA. At the 5’ end, a set of 6 fluorescently labeled “barcodes” is placed, and at the 3’ end, a “capture probe” with biotin is placed. One set allows for simultaneous determination of 800 miRNAs in 12 samples.

Due to the procedure used, the cDNA synthesis and amplification stages were omitted, which allows us to reduce the probability of laboratory error. The results were read out on a NanoString nCounter scanner. The first stage of the analysis was the hybridization of individual miRNAs with specific probes, and the next was the purification and placing of hybridized samples on a specially standardized plate. The last stage was reading of the obtained results. The method allowed for the exact number of miRNA copies to be specified in each sample.

### RT-PCR Validation

Validation of the results was carried out on the same group of patients (24 women in the NGT control group and 24 in GDM study group). To validate the results, the real-time PCR method was used. In the first step, reverse transcription was performed to transcribe miRNA to cDNA using the miRCURY LNA RT Kit (Qiagen, Germany) in accordance with the manufacturer’s procedure on a C1000 Touch Thermal Cycler (Bio-Rad Laboratories, Hercules, USA). Subsequently, we performed RT PCR reaction using the miRCURY LNA SYBR Green PCR Kit (Qiagen, Germany) and specific primers for each of the analyzed miRNAs (Qiagen, Germany) on a LightCycler 480 thermal cycler (Roche Diagnostics Ltd, Switzerland). Expression of circulating miRNAs was evaluated using miR-103a-3p as an endogenous control gene. All samples were assayed in duplicate, and the comparative Ct method was used to calculate the relative changes in gene expression.

### Data Analysis

Analysis of raw miRNA data obtained using NanoString technology was performed in nSolver software version 4.0. Data were normalized by the average geometric mean of the top 100 probes detected. The miRNAs’ expression values in RT-PCT validation were calculated based on the ΔΔCT method. The differences in miRNA expressions between groups were calculated by the Mann-Whitney U test using Statistica 13 for Microsoft Software (StatSoft Inc., Tulsa, USA). The relationships between variables were tested using the Spearman rank correlation coefficient. Results were considered statistically significant with p-value less than 0.05.

## Results

### Characteristics of the Groups Studied

The clinical characteristics of the studied groups are presented as medians and interquartile ranges (**Tables 1**, **2**). In the 1^st^ trimester of pregnancy, there were no significant differences between groups. Women in both groups were normoglycemic. Most patients had normal pre-pregnancy BMI (n=10 in GDM group and n=11 in NGT group had BMI >25 kg/m^2^ indicating overweight). In the 2^nd^ trimester, groups revealed significant differences in fasted and post-loaded glucose measurements (glucose at 0, 30, 60, and 120 minutes: p=0.0001, p=0.0000, p=0.0000, and p=0.001, respectively). The GDM group had a higher insulin level at 60 minutes (p=0.02), insulin level at 120 minutes (p=0.004), and HOMA-IR (p=0.02). Fasting insulin and insulin after 30 minutes post-loading were also higher in the study group than in the NGT group, but the differences were insignificant. Moreover, the GDM group demonstrated lower ISI_OGTT_ (p=0.002) and lower total cholesterol (p=0.046) than the NGT group.

**Table 1:** Clinical characteristics of groups studied in the 1^st^ trimester.

	NGT	GDM	p-value
**n**	24	24	
**Age (years)**	28 (26-31.5)	26 (24-30.5)	0.36
**Pre-pregnancy BMI (kg/m^2^)**	21.8 (20.0-28.2)	23.5 (21.6-26.8)	0.73
**Gestational age (week)**	11 (10-12)	10 (9.5-11)	0.24
**Fasting glucose (mg/dl)**	86 (84-88)	87.5 (85-90)	0.29
**Fasting insulin (µU/ml)**	10.7 (9.1-12.9)	11.3 (10.2-13.3)	0.25
**HOMA-IR**	2.3 (1.9-2.8)	2.5 (2.1-2.9)	0.18
**HOMA-β**	168.0 (145.5-187.5)	166.5 (146.9-201.0)	0.97
**HbA1c (%)**	5.0 (4.9-5.4)	5.0 (4.9-5.4)	0.98
**Total cholesterol (mmol/l)**	170 (149.5-191.5)	169.5 (156.5-186)	0.81
**HDL-cholesterol (mmol/l)**	81 (69.5-90.5)	73.5 (59.5-84.5)	0.19
**LDL-cholesterol (mmol/l)**	78.2 (64.3-91.6)	81 (64.9-95.3)	0.78
**Triglycerides (mmol/l)**	72 (60.5-109.5)	95.5 (68.5-118)	0.14

Data are shown as medians (interquartile range); The difference between NGT vs GDM group was compared with the Mann-Whitney U-test.

**Table 2:** Clinical characteristics of groups studied in the 2^nd^ trimester.

	NGT	GDM	P value
**n**	24	24	
**Gestational age (week)**	25 (25-26)	25 (25-26)	0.81
**Fasting glucose (mg/dl)**	82.5 (79-85)	92 (84-94)	0.0001
**Glucose 30’ (mg/dl)**	127.5 (121-140)	158 (148-165)	< 0.0001
**Glucose 60’ (mg/dl)**	122 (101.5-141.5)	169 (136.5-184)	< 0.0001
**Glucose 120’ (mg/dl)**	105.5 (86-119)	125.5 (111.5-166)	0.001
**Fasting insulin (µU/ml)**	11.2 (8.6-13.3)	13.4 (10.1-18.2)	0.08
**Insulin 30’ (µU/ml)**	74.2 (60.0-110.9)	80.1 (61.0-137.3)	0.62
**Insulin 60’ (µU/ml)**	80.1 (54.3-107.2)	106.5 (74.2-174.0)	0.02
**Insulin 120’ (µU/ml)**	56.0 (42.0-72.4)	108.8 (60.5-131.0)	0.004
**HOMA-IR**	2.3 (1.7-2.7)	3.0 (2.1-4.4)	0.02
**HOMA-β**	188.3 (168.8-282.0)	191.4 (149.4-240.3)	0.21
**ISI OGTT**	4.4 (3.4-5.4)	2.8 (2.1-3.9)	0.002
**HbA1c (%)**	4.8 (4.7-5.1)	4.9 (4.6-5.1)	0.74
**Total cholesterol (mmol/l)**	267.5 (203-287.5)	238 (188-257)	0.046
**HDL-cholesterol (mmol/l)**	96.5 (85.5-108.5)	85 (69.5-104.5)	0.13
**LDL-cholesterol (mmol/l)**	131 (101.4-171.2)	119.2 (85.0-140.4)	0.12
**Triglycerides (mmol/l)**	138 (120.5-170.5)	159 (135.5-204.5)	0.13

Data are shown as medians (interquartile range); The difference between NGT versus GDM group was compared with the Mann-Whitney U-test.

### Nanostring Profiling

We identified 28 miRNAs with expression that was significantly altered in the GDM group compared to the NGT group (p-value p<0.05). A careful analysis was done while considering not only the p-value, but also the false discovery rate, count ranges, fold change, and standard deviation. The results pointed out miR-16-5p (p=0.07), miR-142-3p (p=0.02), miR-144-3p (p=0.003), and miR-320e (p=0.02) for further validation. Changes in expression of miR-16-5p were not significant, whereas the mean value ranges of the counts were high (GDM=1056.03 versus NGT=756.86) with a wide standard deviation. Considering the method of simultaneous determination of many miRNAs and high count number, we decided to evaluate these molecules in further analysis.

### Validation of the Results

**Figure 1:**
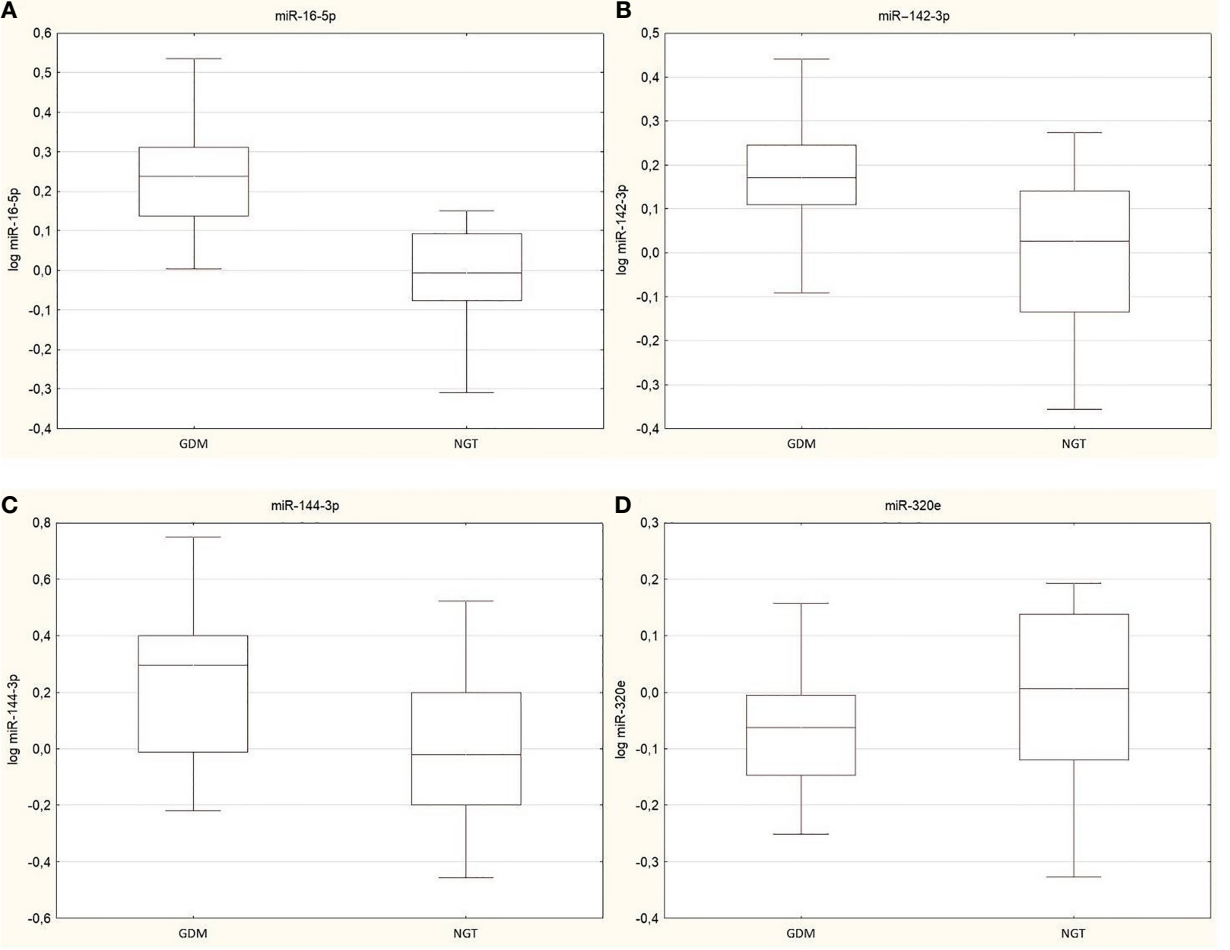
Box plots presented changes in expression of validated miRNAs between GDM group and NGT, **(A)** miR-16-5p (p<0.0001), **(B)/** miR-142-3p (p=0.001), **(C)** miR-144-3p (p=0.003) and **(D)** miR-320e (p=0.16). Data are presented by median indicated by line in each box and interquartile range. Maximum and minimum values are represented by whiskers.

NanoString results were validated by RT-PCR. The fold change of gene expression was calculated using the ΔΔCt method, and then log transformation was used to avoid a non-normal distribution of the results. We obtain confirmation of three miRNAs: miR-16-5p (p<0.0001), miR-142-3p (p=0.001), and miR-144-3p (p=0.003), which were significantly upregulated in the GDM group. No significant difference was observed for miR-320e (p=0.16) (**Figure 1**).

**Figure 2:**
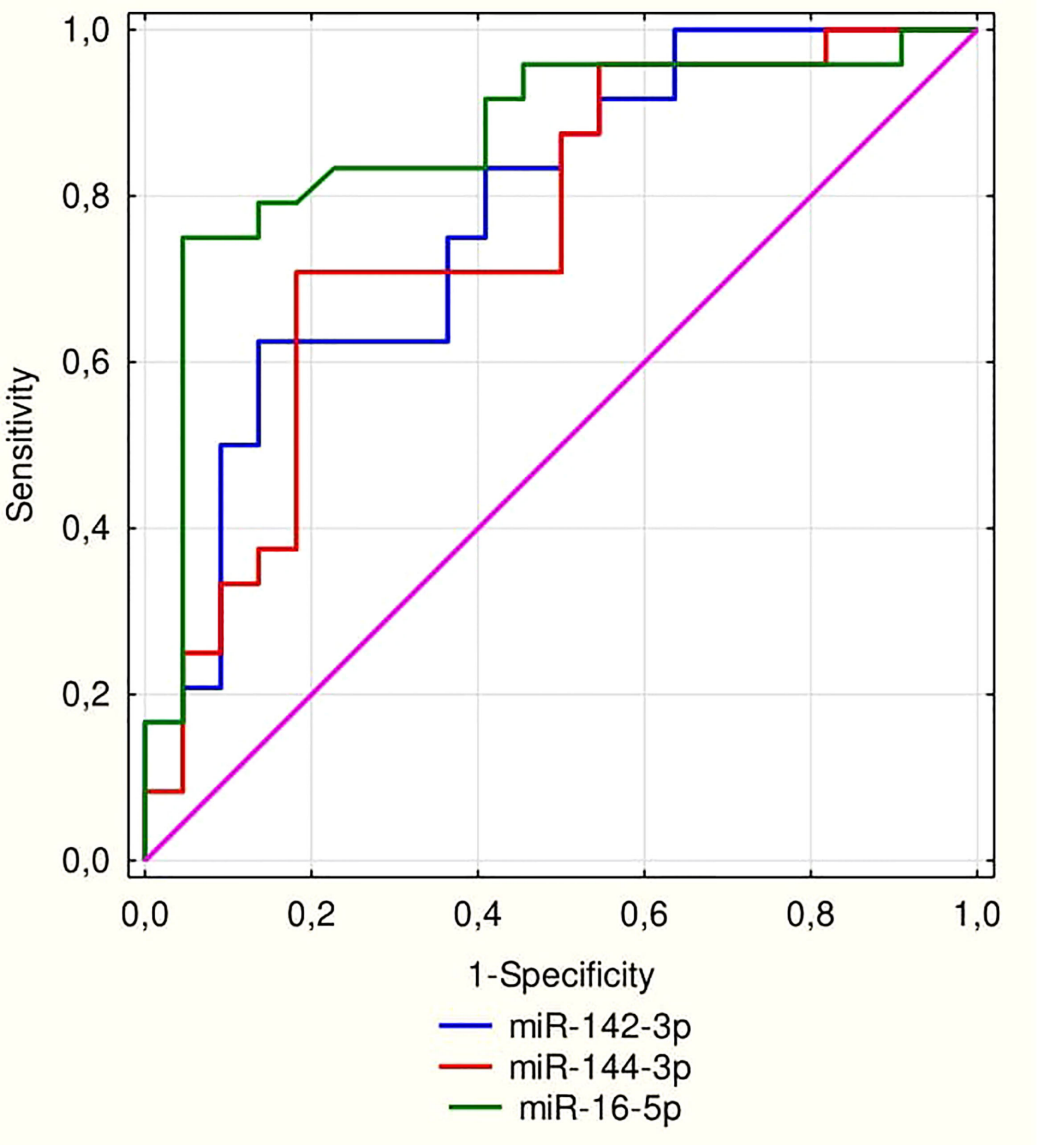
ROC curve for miRNA 16-5p (AUC = 0.868; p < 0.0001), miR-142-3p (AUC = 0.778; p < 0.0001) and for miR-144-3p (AUC = 0.756; p = 0.0004).

ROC curve analysis was performed for significant miRNAs in the 1^st^ trimester of pregnancy as parameters to discriminate those who are at high risk group of developing GDM in the 2^nd^ trimester of pregnancy (**Figure 2**). The AUC for miR-16-5p was 0.868 (95% confidence interval: 0.757–0.98; p<0.0001). AUC was 0.778 (95% confidence interval: 0.644-0.913; p<0.0001) for miR-142-3p, and for miR-144-3p, AUC was 0.756 (95% confidence interval: 0.613-0.898; p=0.0004).

The relationships between prominent molecules’ expressions and other variables were checked. Across the study population, 1^st^-trimester miR-16-5p expression correlated positively with fasting plasma glucose concentration in the 2^nd^ trimester (R=0.56, p<0.05), plasma glucose concentration at 30 minutes post-loading (R=0.43, p<0.05), and HOMA-IR (R=0.36, p<0.05). Its expression negatively correlated with ISI_OGTT_ (R=-0.34, p<0.05). MiRNA-142-3p positively correlated with plasma glucose levels post-loading with indexes as follows: 30 minutes (R=0.35, p<0.05), 60 minutes (R=0.37, p<0.05), and 120 minutes (R=0.36, p<0.05). Furthermore, there were correlations between miR-144-3p and plasma glucose concentration at 30 minutes post-loading (R= 0.41, p<0.05) and the plasma glucose level at 60 minutes post-loading (R=0.42, p<0.05), as well as a negative correlation with ISI_OGTT_ (R=-0.33, p<0.05). Multiple regression analysis confirmed the dependences described except for the association of miR-16-5p and ISI_OGTT_.

## Discussion

Researchers for many years have been trying to find the most ideal GDM biomarker. Among many significant features of the perfect indicator the most relevant is prediction value (24). In case of GDM, the diagnosis nowadays is based on OGTT performed in the second trimester of pregnancy. Considering complications during pregnancy and delivery and a high risk of long-term complications for the child and the mother the GDM a biomarker revealed before changes in the glycemia occur seems to be crucial. Yoffe et al. (25) studied women between 9^th^ and 11^th^ weeks of pregnancy and showed an up-regulation of the miR-223 and miR-23a in plasma of GDM women. Interesting point of view was presented by Wander et al. connection of the miR-21-3p and miR-210-3p with GDM diagnosed in overweight and obese women (26). Lamadrid-Romero et al. (27) showed that miR-183-5p was increased in every trimester in serum collected from women diagnosed with GDM. Simultaneously, the higher expression of miR-125b-3p, miR-200b-3p and miR-1290 were observed in the first trimester of pregnancy.

Our study shows that circulating miR-16-5p is upregulated in women before the onset of GDM, which is consistent with the results obtained by other studies. Zhu et al. (28) conducted studies on women at 16-19 weeks of pregnancy and described five molecules that were upregulated in the GDM group (e.g., miR-16-5p). Other studies reported increased expression of miR-16-5p in serum at 24 -28 weeks of pregnancy (29). Our results show this difference earlier between the 9^th^ and 12^th^ weeks of pregnancy. Moreover, we observed a positive correlation with HOMA-IR, which was also described by Cao et al. (29). Apart from miR-16-5p they described an up-regulation of the miR-17-5p and miR-20a-5p which was not observed in our experiment.

Attempts were made to determine miR-16-5p in leukocytes of women with GDM, but no significant differences were observed (30, 31). It turns out that high miR-16-5p expression also persists after pregnancy and correlates with high cardiovascular risk (32). This indicates that epigenetic changes during GDM are permanent, and women with a history of GDM are predisposed to the development type 2 diabetes (T2D) or cardiovascular disease in the following years (33). Another study revealed increased expression of miR16-5p in overweight women before the 20^th^ week of pregnancy. In contrast to previously cited reports, that study was conducted on European women (34). On the other hand, Martinez-Ibarra et al. demonstrated no significant changes in miR16-5p expression in serum collected in the 2^nd^ trimester from GDM patients compared to NGT (35). A similar result was obtained by scientists from South Africa (36).

Considering that miRNAs could be related to genetic and environmental factors, Sørensen et al. proposed ethnicity as a potential explanation of differences in obtained results (34). Furthermore, they also suggested age, which is a known risk factor for GDM. The idea was supported by the correlation obtained between age and miR-16-5p expression. However, this dependence was not observed in our study.

Available data show that miR-16-5p is one of the most potent regulating molecules in the insulin-signaling pathway. Target genes for miR-16-5p encode insulin receptor substrate (IRS) proteins 1 and 2 and the insulin receptor itself (INSR) (37, 38). These proteins are crucial factors in a proper insulin signaling pathway, and their downregulation results in insulin resistance and metabolic disorders like diabetes. Additionally, miR-16-5p-targeted genes are involved in pancreatic β-cell proliferation and apoptosis (39). Target genes for miR-16-5p that are downregulated in type 2 diabetes are located in not only β-cells on pancreatic islets, but also peripheral blood mononuclear cells (PBMCs), the liver, and skeletal muscle (40).

An experimental study on *Cmah-*null mice showed that diabetic mice have upregulated miR-16-5p (among others) and downregulated IRS1, IRS2, AKT1, and mTOR mRNA (41). As a result of these changes, the crucial pathway in insulin-signaling PI3K-Akt-mTOR is dysregulated (42). Interestingly, Lee et al. (43) demonstrated a decrease in miR-16-5p expression in insulin-resistant skeletal muscle. Moreover, their *in-vitro* study revealed that miR-16-5p is involved in autophagy through controlling Bcl-2 protein synthesis. Also, an overexpression of miR-16-5p was accompanied by decreased mTOR content. Based on these findings, the inhibition of miR-16-5p expression might be important in treatment (44).

There are two reports on miR-142-3p in GDM. However, neither of these studies considers circulating human miR-142-3p. Collares et al. (45) described nine miRNAs (e.g., miR-142-3p) that are upregulated in PBMC obtained from type 1 diabetes (T1D), T2D, and gestational diabetes mellitus. The study did not associate the molecule with a specific gene, but its involvement in diabetes in general was noticeable. A study conducted on GDM-induced mice reported an overexpression of miR-142-3p in the circulating blood and embryonic tissue of GDM mice. Data demonstrated that *in-vitro* up-regulation of miR-143-3p has a positive effect on β-cells by promoting their proliferation, as well as inhibiting apoptosis by blocking the expression of p27, Bax, and caspase-3. In addition, bioinformatic analysis indicated forkhead box protein O1 (FOXO1) as a target gene for miR-142-3p (46). FOXO1 is known as a multifunctional protein, and besides controlling glycogenolysis and gluconeogenesis, it regulates the differentiation of β-cells and promotes their apoptosis (47). This could be a self-protective effect of miR-142-3p.

Escalated expression of miR-142-3p has been described in obese adults as a parameter that is strongly associated with insulin, HOMA-IR, BMI, adiponectin, and leptin levels (48). Similar results were obtained in the case of childhood obesity, which revealed an increased concentration of miR-142-3p and a positive correlation with BMI, fat mass, adipose tissue distribution, and HOMA-IR. Interestingly, during a 3-year follow-up, upregulation in the expression of this molecule was observed solely in the serum of patients whose BMI remained stable or decreased (49). The data showed that the expression of the miR-142-3p may be sex-related.

Overexpression of miR-142-3p in the group of patients with pre-diabetes and diabetes was found only among women (50). The studies mentioned the possibility of age affecting these results because the male group was significantly younger. However, there may also be an influence from the distribution of adipose tissue according to the studies cited. In contrast to our study, Liang et al. showed a decreased expression of miR-142-3p in the serum of T2D patients, and a negative correlation with HOMA-IR was observed (51). In the present study, a positive correlation was revealed between miR142-3p and plasma glucose post-loading.

Another study has shown that miR-144-3p is upregulated in the liver, pancreas, skeletal muscle, adipose tissue, and blood of a diabetic rat model. The result was confirmed in circulating blood obtained from human T2D patients. In addition, a study of pancreatic cells cultured from rats revealed an increased level of miR-144-3p in a high glucose environment, and similar to miR-16-5p, it caused a downregulation of the expression of IRS1 (52). Moreover, the upregulation of miR-144-3p was observed in PBMCs collected from patients with T1D, T2D, and GDM (45). However, Akerman et al. (53) investigated patients with T1D and did not observe an elevation of serum miR-144-3p levels, but there was a positive correlation with islet antigen 2 antibodies (IA2A), indicating a possible relationship with the assessment of those at risk for T1D development.

Upregulated expression and a positive correlation with HOMA-IR of circulating miR-144-3p were observed in a Chinese cohort with impaired fasting glucose (IFG). Furthermore, high miR-144-3p was a predictor of T2D development (51). Interestingly, Wang et al. (54) showed an increased expression of miR-144-3p in T2D patients but solely in a Swedish population, not in patients from Iraq. Thus, this report confirms the contribution of environmental factors to epigenetic changes mentioned above. In a meta-analysis, Zhu and Leung (55) selected eight molecules as potential biomarkers of T2D, including miR-142-3p and miR-144-3p.

In summary, we found significantly upregulated expression of miR-16-5p, miR-142-3p, and miR-144-3p in the serum of patients in their 1^st^ trimester of pregnancy who suffered from GDM diagnosed in the 2^nd^ trimester. NanoString technology allowed us to study a wide panel of miRNA profiles. Considering the research on miRNAs, a strong point of our experiment was the large number of patients in the studied groups. Although our findings are limited by the validation using the same group of women, our observations strongly suggest that changes taking place in the miRNA profile occur earlier than changes in glucose levels, and research on the more sensitive and specific biomarkers of GDM should be continued.

## Data Availability Statement

The raw data supporting the conclusions of this article will be made available by the authors, without undue reservation.

## Ethics Statement

The studies involving human participants were reviewed and approved by Ethics Committee Medical University of Bialystok. The patients/participants provided their written informed consent to participate in this study.

## Author Contributions

Conceptualization: IJ and MK. Methodology: MN, IJ, IS and AB. Formal analysis: MZK and AK. Writing—original draft preparation: IJ and MK. Writing—review and editing: AJK and JS. Supervision: JS and AJK. All authors contributed to the article and approved the submitted version.

## Funding

The study was supported by funds from Medical University of Białystok, Poland SUB/1/DN/20/002/1129, SUB/1/DN/19/001/1129.

## Conflict of Interest

The authors declare that the research was conducted in the absence of any commercial or financial relationships that could be construed as a potential conflict of interest.

## Publisher’s Note

All claims expressed in this article are solely those of the authors and do not necessarily represent those of their affiliated organizations, or those of the publisher, the editors and the reviewers. Any product that may be evaluated in this article, or claim that may be made by its manufacturer, is not guaranteed or endorsed by the publisher.
